# A systematic review and meta-analysis of thoracic epidural analgesia versus other analgesic techniques in patients post-oesophagectomy

**DOI:** 10.1186/s13741-024-00437-0

**Published:** 2024-07-23

**Authors:** Duncan Macrosson, Adam Beebeejaun, Peter M. Odor

**Affiliations:** 1 Department of Anaesthesia and Perioperative Medicine, University College London Hospitals NHS Foundation Trust, London, England; 2 University College London, London, England

**Keywords:** Thoracic epidural, Oesophagectomy, Analgesia, Postoperative pulmonary complications, Meta-analysis, Systematic review

## Abstract

**Background:**

Oesophageal cancer surgery represents a high perioperative risk of complications to patients, such as postoperative pulmonary complications (PPCs). Postoperative analgesia may influence these risks, but the most favourable analgesic technique is debated. This review aims to provide an updated evaluation of whether thoracic epidural analgesia (TEA) has benefits compared to other analgesic techniques in patients undergoing oesophagectomy surgery. Our hypothesis is that TEA reduces pain scores and PPCs compared to intravenous opioid analgesia in patients post-oesophagectomy.

**Methods:**

Electronic databases PubMed, Excerpta Medica Database (EMBASE) and Cochrane Central Register of Controlled Trials (CENTRAL) were searched for randomised trials of analgesic interventions in patients undergoing oesophagectomy surgery. Only trials including thoracic epidural analgesia compared with other analgesic techniques were included. The primary outcome was a composite of respiratory infection, atelectasis and respiratory failure (PPCs), with pain scores at rest and on movement as secondary outcomes. Data was pooled using random effect models and reported as relative risks (RR) or mean differences (MD) with 95% confidence intervals (CIs).

**Results:**

Data from a total of 741 patients in 10 randomised controlled trials (RCTs) from 1993 to 2023 were included. Nine trials were open surgery, and one trial was laparoscopic. Relative to intravenous opioids, TEA significantly reduced a composite of PPCs (risk ratio (RR) 3.88; 95% confidence interval (CI) 1.98–7.61; *n* = 222; 3 RCTs) and pain scores (0–100-mm visual analogue scale or VAS) at rest at 24 h (*MD* 9.02; 95% *CI* 5.88–12.17; *n* = 685; 10 RCTs) and 48 h (*MD* 8.64; 95% *CI* 5.91–11.37; *n* = 685; 10 RCTs) and pain scores on movement at 24 h (*MD* 14.96; 95% *CI* 5.46–24.46; *n* = 275; 4 RCTs) and 48 h (*MD* 16.60; 95% *CI* 8.72–24.47; *n* = 275; 4 RCTs).

**Conclusions:**

Recent trials of analgesic technique in oesophagectomy surgery are restricted by small sample size and variation of outcome measurement. Despite these limitations, current evidence indicates that thoracic epidural analgesia reduces the risk of PPCs and severe pain, compared to intravenous opioids in patients following oesophageal cancer surgery. Future research should include minimally invasive surgery, non-epidural regional techniques and record morbidity, using core outcome measures with standardised endpoints.

**Trial registration:**

Prospectively registered on PROSPERO (CRD42023484720).

## Background

Oesophagectomies are considered major and complex surgery with significant postoperative pain and high postoperative complication rates which can decrease long-term survival (Booka et al. 2018). Epidural analgesia is often considered the gold standard form of postoperative analgesia for this surgery (Low et al. 2018). Epidurals have been shown to reduce postoperative pain scores and some postoperative complications such as respiratory failure in major abdominal surgery but with uncertain replication in oesophageal cancer surgery (Pirie et al. 2020; Rigg et al. 2002). Pulmonary complications are of interest as they are one of the most common postoperative complications, especially in high-risk open abdominal surgery; they predict long- and short-term health outcomes, admission to critical care and hospital length of stay (Booka et al. 2018; Odor et al. 2020).

Although epidural analgesia may potentially improve outcomes, contraindications include patient refusal, anticoagulant use or a patient’s pre-existing anatomical or neurological issues. Epidurals are associated with complications such as urinary retention, hypotension and partial or complete failure and rarer complications such as neurological damage (8.2–17.4 cases of permanent nerve damage per 100,000 patients receiving epidural analgesia) (Cook et al. 2009). Postoperative management of epidural analgesia also represents a higher resource requirement (Holtz et al. 2022). Recent evidence has suggested minimally invasive oesophagectomy surgery is gaining in popularity compared to open oesophagectomy surgery (Mann et al. 2020). Existing evidence from colorectal surgical data shows epidural analgesia achieves superior pain relief compared to opioid analgesia for open surgery, but not for less invasive (laparoscopic) surgery (Borzellino et al. 2024; Turi et al. 2024). Therefore, as minimally invasive oesophagectomy surgery increases in frequency, epidural analgesia may in turn become less beneficial. Finally, other regional techniques such as paravertebral and erector spinae catheters have been gaining favour in recent years, having many of the benefits of epidural analgesia but with a more favourable side effect profile, although randomised clinical trials are lacking (Feenstra et al. 2023). Many of these factors may result in a reduction in the use of epidural analgesia for postoperative pain management (Pirie et al. 2020).

Two previous meta-analyses compared analgesic techniques in oesophagectomies in 2017 and 2018 but found a paucity of prospective trials to compare. Regarding epidural analgesia versus intravenous opioid analgesia, Visser et al. (2017) observed no significant difference in pain scores at 24 and 48 h postoperatively, and Hughes et al. (2018) observed no significant difference in rest pain postoperatively (Visser et al. 2017; Hughes et al. 2018). Both reviews concluding that no benefit could be shown by epidurals regarding postoperative pulmonary complications (PPCs). Since these reviews, further relevant randomised trials have been published (Xu et al. 2023; Zhu et al. 2020; Li et al. 2019; Wang et al. 2019).

During the completion of this review, a network meta-analysis evaluating analgesic strategies post-oseophagectomy by Ramjit et al. (2024) was published showing an increase in postoperative forced vital capacity; a reduction in pain scores, opioid consumption, intensive care unit stay and time to extubation in thoracic epidural analgesia (TEA) versus systemic opioids (Ramjit et al. 2024). The review did not find enough data to analyse morbidity including postoperative pulmonary complications.

Our primary aim was to evaluate whether TEA reduced respiratory morbidity versus other analgesic techniques following oesophagectomy surgery, with a secondary objective to compare analgesic outcomes.

## Methods

This review protocol was prospectively registered on PROSPERO (CRD42023484720) and followed guidance from the Preferred Reporting Items for Systematic reviews and Meta-Analysis (PRISMA) statement 2020 (Page et al. 2020).

The review question was as follows: “In adult patients undergoing elective oesophagectomy, does thoracic epidural analgesia influence postoperative pulmonary complications in comparison to other analgesic techniques?” We used the framework of PICOS (Population, Intervention, Comparison, Outcomes and Study design). Participants included adult patients undergoing elective oesophagectomy. Thoracic epidural analgesia was the comparator group. The intervention groups included any other form of analgesia such as intravenous opioids or other regional techniques. Study design was restricted to randomised clinical trials only.

### Primary outcome

The primary outcome was a composite of postoperative pulmonary complications (PPCs), including respiratory infection, respiratory failure and atelectasis within 30 days of surgery. Standard diagnostic criteria were based upon the European Perioperative Clinical Outcomes (EPCO) consensus statement (Jammer et al. 2015). However, as the review search dates included a period before the most recent consensus definitions of PPCs, we categorised explicit descriptions of PPCs in each trial according to closeness of match to the EPCO definitions. Where a composite PPC was not reported, we contacted corresponding authors via email to request additional information, including primary data.

### Secondary outcomes

Secondary outcomes included resting and dynamic 24- and 48-h pain scores measured with a 100-mm visual analogue scale (VAS), technical failure, postoperative nausea and vomiting (PONV) and length of hospital stay. Pain not only is important for humane reasons but also it slows progress towards enhanced recovery targets and can lead to further postoperative complications (Low et al. 2018). Technical failure was assessed because epidurals have a high rate of failure (27–32%) (Hermanides et al. 2012). This is at odds with the most common intervention group of intravenous opioid analgesia, which has virtually no failure rate. Technical failure can be defined as insufficient epidural analgesia which requires removal or switch of analgesic regimen and also includes accidental catheter dislodgement (Hermanides et al. 2012). PONV is included as it is a well-known side effect of opioid analgesia; it can be defined as the 24-h incidence of postoperative nausea or vomiting, as this is commonly reported in trials and the most clinically relevant time interval (Dolin and Cashman 2005). Finally, length of hospital stay is a well-established and important perioperative outcome, measured in time (hours) from admission to discharge.

### Search strategy

We searched PubMed, Embase, and CENTRAL databases, using a combination of relevant keywords and medical subject heading terms for oesophagectomy surgery and epidural analgesia. Search limits were applied to restrict results to RCTs published from 1 January 2013 to 31 December 2023. We included all randomised controlled trials of adult (age ≥ 18 years) patients undergoing elective oesophagectomy surgery in which one group received postoperative TEA. Intraoperative TEA was not included as all patients undergo general anaesthesia; thus, pain control and its sequelae are more relevant for the postoperative period. The full search strategy is detailed in Appendix. No language restrictions were placed on eligible studies.

### Study selection

**Fig. 1:**
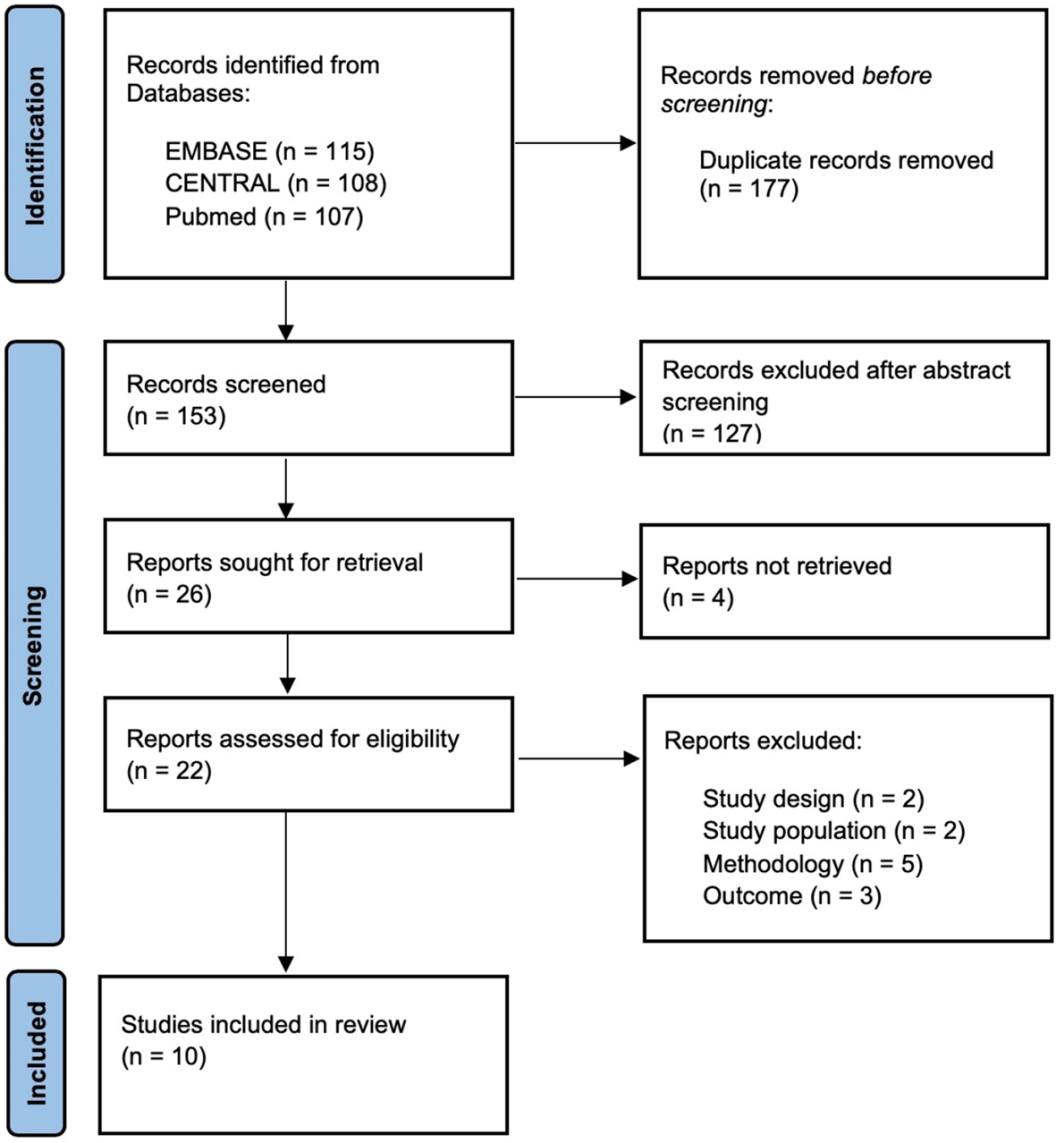
PRISMA flow diagram

After de-duplication, the primary author screened titles and abstracts against the inclusion criteria to identify potentially relevant papers. One researcher was used at this stage who erred on the side of over-inclusion. The second stage involved full-text review of all potentially eligible studies by two authors and recording the reason for the exclusion of a paper (Fig. 1).

### Data extraction

One author extracted data from the selected publications using a pre-piloted data abstract tool. All data was checked by a second reviewer. Information included is as per the study characteristic tables below. Data not reported in the studies was recorded as “NR” (not reported), and non-applicable data was recorded as “N/A”. WebPlotDigitizer was used to estimate these numerical scores and standard deviations from graphical data (Rohatgi 2022). Risk-of-bias assessment was completed after data extraction. Two authors individually evaluated the methodological quality of all articles using the Cochrane risk-of-bias assessment tool version 2 (Higgins et al. 2023).

### Data synthesis

Meta-analysis was performed on any primary or secondary outcome included by more than one study. For the dichotomous outcomes of PPCs and PONV, incidences of outcomes per group were extracted from each study to allow a pooled meta-analysis of risk ratio estimates with 95% confidence intervals. For the continuous outcomes of 24- and 48-h pain scores for resting and dynamic pain, mean scores and standard deviations for both groups in each study were extracted to allow a pooled meta-analysis of mean difference estimates with 95% confidence intervals.

Where results were presented in included trials as mixed data of median (IQR) and mean (SD), we converted to mean and standard deviation throughout, to enable pooled comparison. Standard deviations for pain scores were imputed for one trial that did not report, by combining the mean standard deviations of other trials (Fares et al. 2014). One trial measured postoperative pain scores twice a day; these morning and afternoon scores were combined to give a single mean and standard deviation for each day (Flisberg et al. 2001). Two trials had four groups of participants we combined into two groups according to the method of postoperative analgesia (Zhu et al. 2020; Li et al. 2019). Standard deviations were computed using an online calculator (StatsToDo) decomposing the mean and standard deviations of two groups into one single group (CombineMeanSD. 2023; Altman 2000).

All meta-analyses were performed using RevMan (Cochrane and Collaboration 2024). Inverse variance was used as the statistical method for both dichotomous and continuous outcomes. Statistical heterogeneity was assessed by using both the *I*^2^ and *χ*^2^ tests. A random effects model was adopted due to the clinical and methodological diversity between trials. Formal meta-analyses were not possible for other outcomes including technical failure and length of hospital stay due to insufficient data; therefore, this data is presented in tabulated form and/or narratively appraised.

## Results

### Description of included studies

A total of 330 publications were found over three databases. After filtering for eligibility criteria, 10 randomised trials with 741 patients over 5 countries were included (Table 1).

**Table 1:** Study information

Author and Year	Country	Design	Randomisation	Allocation concealment	Source of funding	Number	Surgical approach
Flisberg et al. 2001	Sweden	RCT	NR	NR	University and Society of Medicine grants	33	Open
Yokoyama et al. 2005	Japan	RCT	NR	NR	NR	30	Open
Fares et al. 2014	Egypt	RCT	Computer generated	Opaque envelopes	None	30	Open
Wang et al. 2017	China	RCT	Random number table	NR	NR	80	Open
Wang et al. 2019	China	RCT	Computer generated	Opaque envelopes	Innovation and Science Foundation grants	40	Open
Liu and Wang 2015	China	RCT	NR	NR	NR	60	Open
Li 2019	China	RCT	Computer generated	Opaque envelopes	Science Foundation and educational grants	100	Open
Zhu et al. 2020	China	RCT	Computer generated	NR	NR	120	Open
Maghsoudloo et al. 2023	Iran	RCT	Based on odd/even surgical date	NR	NR	80	Open
Xu et al. 2023	China	RCT	Computer generated	Opaque envelopes	Cancer, Science Foundation and research grants	168	Laparoscopic

*RCT* randomised controlled trial, *NR* not reported

The details of inclusion and exclusion criteria varied between trials, with many excluding comorbid patients based on ASA grade or individual diseases. Only one trial used a laparoscopic technique for surgery; all others were open surgery. Three trials recorded PPCs, and only two trials recorded length of hospital stay and PONV. However, all trials recorded some form of pain score at 24 and 48 h (Table 2).

**Table 2:** Inclusion and exclusion criteria and primary and secondary trial outcomes matching systematic review outcomes

Year and author	Inclusion criteria	Exclusion criteria	Primary outcomes	Secondary outcomes
Flisberg et al. 2001	ASA 1–3, thoracoabdominal oesophagectomy	NR	Nil	Pain scores at 24 and 48 h
Yokoyama et al. 2005	ASA 1–2, radical oesophagectomy, oesophageal cancer	Preoperative steroid/NSAID	Nil	Pain scores at 24 and 48 h
Fares et al. 2014	ASA 1–2, age 20–60 years, Ivor Lewis oesophagectomy	NYHA 3–4, chronic obstructive pulmonary disease, CKD, Child–Pugh B liver failure or worse, bleeding diathesis. Preoperative steroid	Pulmonary complications	Pain scores at 24 and 48 h
Liu and Wang 2015	*ASA* 1–2, age 40–65 years, 45–70 kg, radical oesophagectomy and thoracotomy	Preoperative radiotherapy or chemotherapy. Endocrine/metabolic diseases, antiplatelet/anticoagulant drugs. Perioperative blood transfusion	Nil	Pain scores at 24 and 48 h
Wang et al. 2017	*ASA* 1–2, age 18–65 years, BMI 18–30	Heart/liver/kidney/electrolyte/neuromuscular/immune/endocrine disorder. Postoperative mechanical ventilation or secondary surgery during study	Nil	Pain scores at 24 and 48 h, PONV, LOS
Wang et al. 2019	Elective radical oesophagectomy	ASA 4, preoperative opioids/NSAIDs/immunosuppressive drugs. Cardiovascular/immune/endocrine disease	Nil	Pain scores at 24 and 48 h
Li 2019	ASA 1–2, age 40–65 years, 45–80 kg, thoracoabdominal oesophagectomy, gastro-oesophageal carcinoma	Preoperative chemo/radiotherapy, cardiovascular/autoimmune/endocrine/metabolic/coagulation/kidney/liver disorder. Preoperative opioid/NSAID/blood transfusion. Postoperative mechanical ventilation	Nil	Pain scores at 24 and 48 h, PONV
Zhu et al. 2020	ASA 1–2, thoracotomy	Preoperative chemo/radiotherapy/opioid/immunosuppressant/steroid. Autoimmune/endocrine/metabolic diseases. Abnormal liver and kidney function tests. Perioperative blood transfusion	Nil	Pain scores at 24 and 48 h
Maghsoudloo et al. 2023	20–80 years, oesophagectomy	ASA 3 or higher, coagulation/severe haemodynamic/severe movement/chest/neurological disorders, substance abuse	Pulmonary complications	Pain scores at 24 and 48 h
Xu et al. 2023	> 17 years, thoraco-laparoscopic (McKeown) oesophagectomy	Open oesophagectomy, chronic pain with opioid use, not suitable for one lung ventilation, prior lung surgery	Pulmonary complications	Pain scores at 24 and 48 h, LOS

*ASA* American Society of Anesthesiologists physical status grade, *y* years, *BMI* body mass index, *NSAID* nonsteroidal anti-inflammatory drug, *NYHA* New York Heart Association heart failure grade, *CKD* chronic kidney disease, *PONV* postoperative nausea and vomiting, *LOS* length of stay in hospital

Patient characteristics included a mean age of 59.9 years, mean BMI of 22.7 and a proportion of 75.3% males (Table 3). All trials compared TEA to intravenous opioids, with only one trial including a third group of paravertebral and transversus abdominis plane blocks. Regional and intravenous drug regimens varied (Table 4). PPCs, rest and dynamic pain scores are displayed in Tables 5, 6 and 7 respectively and PONV and length of hospital stay in Tables 8 and 9.

**Table 3:** Patient characteristics

Year and author	Analgesic modality	*N*	Age (years)	Male/female	ASA ½/3	Weight (kg)	Height (cm)	BMI
Flisberg et al. 2001	TEA	18	61	15/3	5/10/3	77	172	26.0
	IV	15	68	9/6	1/12/2	65	170	22.5
Yokoyama et al. 2005	Bilevel TEA	15	60 + /– 8	13/2	NR	61 + /– 9	162 + /– 10	23.2
	IV	15	62 + /– 9	12/3	NR	60 + /– 7	161 + /– 8	23.2
Fares et al. 2014	TEA	15	53 + /– 10	12/3	9/6/0	NR	NR	23.9 + /– 1.5
	IV	15	59 + /– 6	11/4	8/7/0	NR	NR	22.1 + /– 3.3
Liu and Wang 2015	TEA	30	56 + /– 8	25/9	13/17/0	53 + /– 14	NR	NR
	IV	30	54 + /– 11	23/7	11/19/0	55 + /– 13	NR	NR
Wang et al. 2017	TEA	40	56 + /– 7	24/16	12/28/0	63 + /– 7	165 + /– 5	23.1
	IV	40	59 + /– 4	26/14	14/26/0	64 + /– 7	166 + /– 7	23.2
Wang et al. 2019	TEA	20	56 + /– 14	NR	NR	62 + /– 10	168 + /– 8	22.0
	IV	20	56 + /– 14	NR	NR	60 + /– 9	165 + /– 8	22.0
**Li 2019**	TEA	50	57 + /– 5	42/8	NR	NR	NR	22 + /– 4
	IV	50	58 + /– 5	42/8	NR	NR	NR	23 + /– 4
Zhu et al. 2020	TEA	60	62 + /– 7	49/11	23/37	61 + /– 7	NR	NR
	IV	60	61 + /– 7	45/15	19/41	61 + /– 6	NR	NR
Maghsoudloo et al. 2023	TEA	40	63 + – 8	17/23	NR	NR	NR	NR
	IV	40	63 + /– 9	24/16	NR	NR	NR	NR
Xu et al. 2023	TEA	56	62	48/8	0/54/2	NR	NR	22.4
	IV	56	61	48/8	0/53/3	NR	NR	22.0
	PVB + TAP	56	63	43/13	1/46/9	NR	NR	22.2

**Table 4:** Analgesic regimens

Year and author	Analgesic modality	*N*	Analgesic location	Drug regimen	Additional analgesia
Flisberg et al. 2001	TEA	18	T6–12	4 ml/h 0.25% bupivacaine + 0.125 mg/ml morphine	4–6 ml 0.25% bupivacaine clinician bolus + / − clinician SC morphine
	IV	15		0.5–4 mg/h IV morphine	0.5–2 mg IV PCA morphine/15-min lockout + /– clinician SC morphine
Yokoyama et al. 2005	Bilevel TEA	15	T34 + T10–11	4 ml/h 0.2% ropivacaine + 4 mcg/ml fentanyl	5-ml 0.2% ropivacaine clinician bolus
	IV	15		1 mg/h morphine IV	2.5-mg IV PCA bolus^a^
Fares et al. 2014	TEA	15	T5–7	0.1 ml/kg/h 0.125% bupivacaine + 5 mcg/ml fentanyl	1-mg IV PCA morphine/5-min lockout
	IV	15		1-mg morphine IV PCA/5-min lockout	NR
Liu and Wang 2015	TEA	30	T7–8	4-ml load + 4 ml/h of 0.1% ropivacaine	4-ml PCEA bolus/40-min lockout
	IV	30		5-ml load + 1 ml/h IV of 80-ml normal saline with 800-mg tramadol + 100-mg flurbiprofen	2-ml IV PCA bolus/15-min lockout
Wang et al. 2017	TEA	40	T7–8	3–4 ml/h 0.125% bupivacaine + 20 mcg/ml morphine	3–4 ml PCEA bolus^a^
	IV	40		0.6–1 mg/h morphine	2–3 mg IV PCA bolus^a^
Wang et al. 2017	TEA	20	NR	3 ml/h 0.125% ropivacaine + 0.4 mcg/ml sufentanil	3 ml/15-min lockout PCEA bolus
	IV	20		0.03 mcg/kg/h sufentanil + 0.5 mg/ml flurbiprofen at 3 ml/h	3-ml PCA bolus/15-min lockout (sufentanil + flurbiprofen)
**Li 2019**	TEA	50	T7–8	0.125% ropivacaine + 2 mcg/ml fentanyl at 5 ml/h	2-ml PCEA bolus/15-min lockout
	IV	50		6 mcg/kg fentanyl + 12 mg/kg tramadol in 100 ml at 2 ml/h	2-ml IV PCA bolus/15-min lockout
Zhu et al. 2020	TEA	60	T4–6	2 ml/h of 100-ml normal saline with 200–300 mcg fentanyl, 150-mg ropivacaine and 5-mg droperidol	0.5-ml PCEA bolus/15-min lockout
	IV	60		2 ml/h of 100-ml normal saline with 15 mcg/kg fentanyl	2-ml IV PCA bolus/15-min lockout
Maghsoudloo et al. 2023	TEA	40	T6–8	4 ml/h 0.125% bupivacaine + 1 ml/h PCEA up to max 2 ml/h	3-mg IV PCA morphine bolus^a^
	IV	40		10 mcg/kg/h morphine + ketorolac 120 mg/day	3-mg IV PCA morphine bolus^a^
Xu et al. 2023	TEA	56	T6–9	2 ml/h 0.15% ropivacaine + 0.12 mg/kg morphine in 100 ml	4-ml PCEA bolus/60-min lockout
	IV	56		1 mg/h IV PCA oxycodone	2-mg IV PCA bolus/5-min lockout
	PVB + TAP	56	T4–7	PVB 15-ml 0.33% ropivacaine at each level. TAP 20-ml 0.25% ropivacaine. Both intraoperative single-shot blocks	1 mg/h IV oxycodone + 2-mg IV PCA bolus 5-min lockout

*N* number, *IV* intravenous, *TEA* thoracic epidural analgesia, *PCA* patient-controlled analgesia, *PCEA* patient-controlled epidural analgesia, *SC* subcutaneous, *PVB* paravertebral block, *TAP* transversus abdominis plane block. ^a^No lockout time reported

**Table 5:** Postoperative pulmonary complications (PPCs)

Year and author	Analgesic modality	*N*	Pulmonary complications
Fares et al. 2014	TEA	15	3
	IV	15	12
Maghsoudloo et al. 2023	TEA	40	0
	IV	40	5
Xu et al. 2023	TEA	56	5
	IV	56	17
	PVB + TAP	56	7

**Table 6:** Pain scores at rest (+ / − standard deviations)

Year and author	Analgesic modality	*N*	Pain score 24-h rest	Pain score 48-h rest	Pain score method
Flisberg et al. 2001	TEA	18	18.75 + / − 5.23	13.5 + / − 3.80	VAS
	IV	15	15.75 + / − 4.57	12.5 + / − 4.42	
Yokoyama et al. 2005	Bilevel TEA	15	7 + / − 7	7 + / − 7	Box scale
	IV	15	12.5 + / − 8.5	11 + / − 7	
Fares et al. 2014	TEA	15	9 + / − 6.82	7 + / − 5.34	VAS
	IV	15	25 + / − 7.57	25 + / − 6.94	
Liu and Wang 2015	TEA	30	6.5 + / − 1.5	5.7 + / − 1.2	VAS
	IV	30	17.7 + / − 4.1	15.8 + / − 3.4	
Wang et al. 2017	TEA	40	29 + / − 2	25 + / − 2	VAS
	IV	40	43 + / − 4	32 + / − 4	
Wang et al. 2019	TEA	20	15.7 + / − 5.9	21.5 + / − 4.8	VAS
	IV	20	24.7 + / − 7.3	31.5 + / − 5.4	
**Li 2019**	TEA	50	22.5 + / − 7.44	21 + / − 5.83	VAS
	IV	50	33 + / − 8.67	30.5 + / − 8.67	
Zhu et al. 2020	TEA	60	17.5 + / − 9	13 + / − 6.5	VAS
	IV	60	26 + / − 7	22.5 + / − 6	
Maghsoudloo et al. 2023	TEA	40	38 + / − 11	28 + / − 7	VAS
	IV	40	41 + / − 11	31 + / − 9	
Xu et al. 2023	TEA	56	4.3 + / − 7	2.6 + / − 5.8	VAS
	IV	56	18.5 + / − 9.5	19 + / − 11	
	PVB + TAP	56	6 + / − 9	6.2 + / − 9

**Table 7:** Pain scores on movement (+ / − standard deviations)

Year and author	Analgesic modality	*N*	Pain score 24-h dynamic	Pain score 48-h dynamic	Dynamic pain score
Flisberg et al. 2001	TEA	18	33.5 + / − 8.74	37.75 + / − 7.37	Movement to sitting position
	IV	15	35 + / − 9.24	43 + / − 7.85	
Fares et al. 2014	TEA	15	28 + / − 9.96	27 + / − 8.78	NR
	IV	15	48 + / − 11.32	48 + / − 10.38	NR
Li et al. 2019	TEA	50	28 + / − 7.13	23.5 + / − 5.67	On active coughing
	IV	50	40.5 + / − 9.72	38.5 + / − 9.29	
Xu et al. 2023	TEA	56	10 + / − 14	11.5 + / − 13	NR
	IV	56	36 + / − 15	37 + / − 14	NR
	PVB + TAP	56	16.5 + / − 14	19 + / − 14	NR

**Table 8:** Postoperative nausea and vomiting

Year and author	Analgesic modality	*N*	Postoperative nausea and vomiting
Wang et al. 2017	TEA	40	13
	IV	40	14
**Li 2019**	TEA	50	16
	IV	50	5
Xu et al. 2023	TEA	56	0
	IV	56	2
	PVB + TAP	56	0

**Table 9:** Length of hospital stay

Year and author	Analgesic modality	*N*	Length of hospital stay (days)
Flisberg et al. 2001	TEA	18	17 (range 9–59)
	IV	15	16 (range 8–44)
Xu et al. 2023	TEA	56	15
	IV	56	15
	PVB + TAP	56	14

**Fig. 2:**
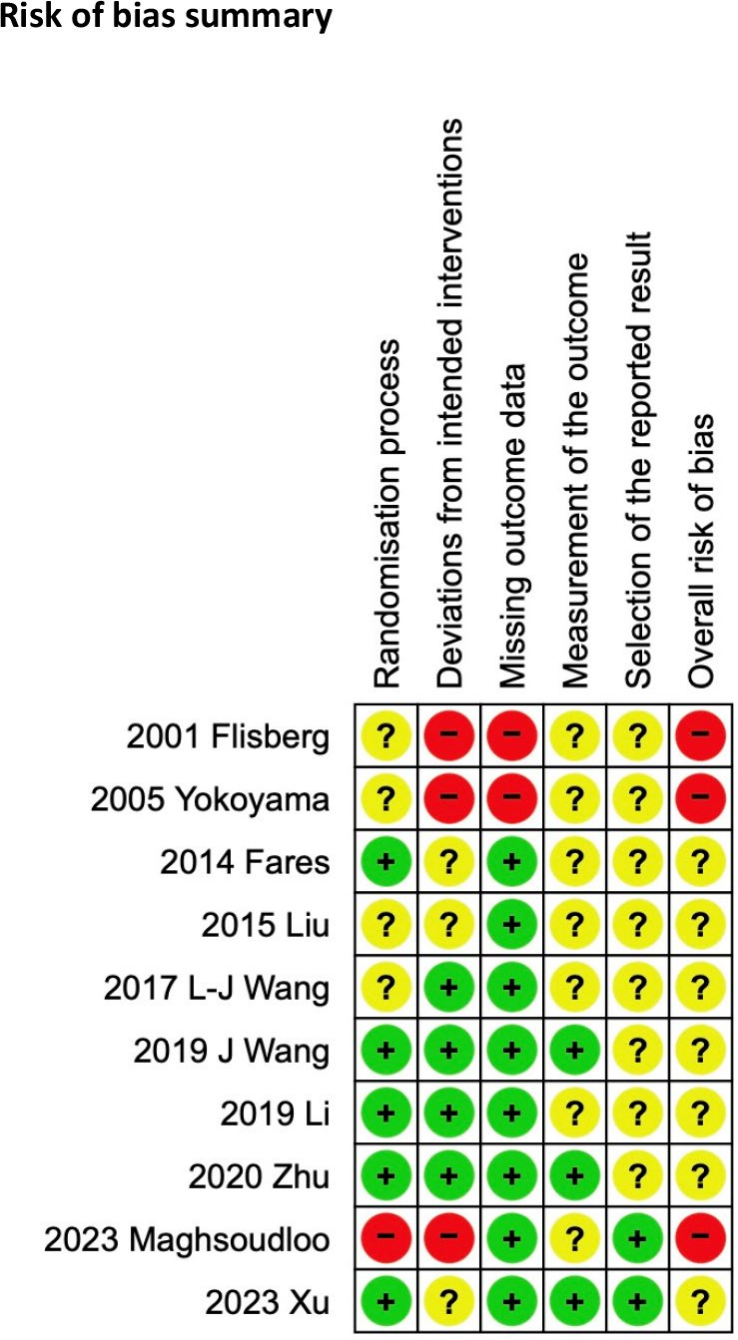
Risk-of-bias summary

Regarding overall risk of bias, seven trials were judged as having some concerns, with three being judged as high risk (Fig. 2). Some trials did not mention their randomisation or allocation concealment method. Nearly all trials did not blind their assessors, accounting for zero trials able to be judged as a low risk of bias overall.

### Postoperative pulmonary complications (PPCs)

**Fig. 3:**
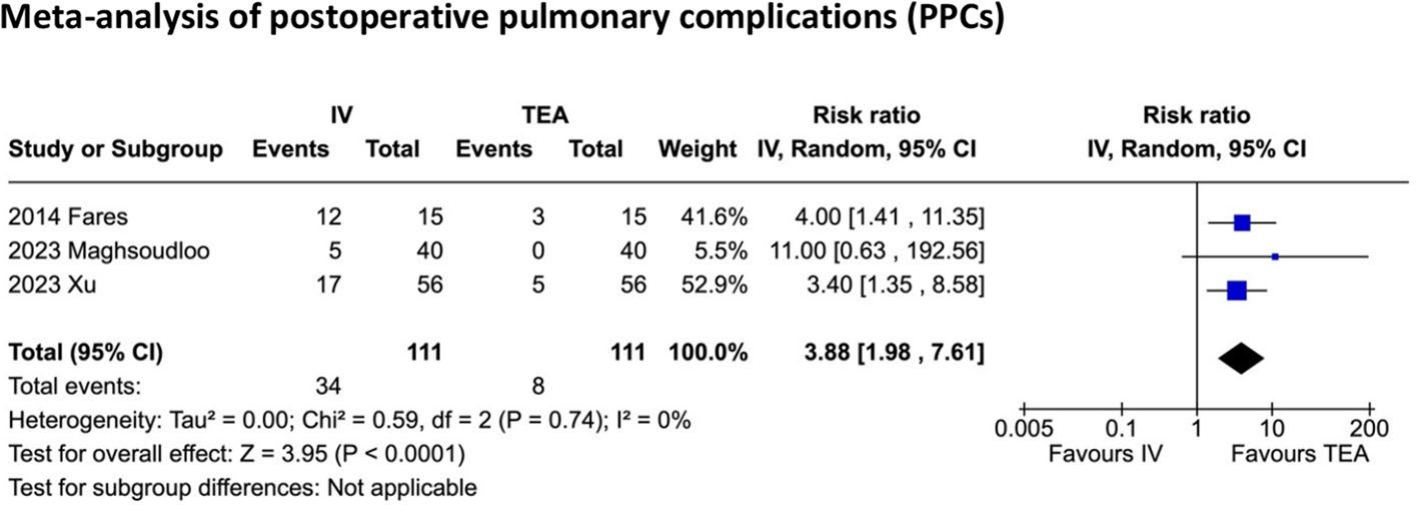
Meta-analysis of postoperative pulmonary complications (PPCs)

Regarding the primary outcome of PPCs within 30 days of surgery, one trial (Xu et al. 2023) used the EPCO definition (Jammer et al. 2015). Another trial (Maghsoudloo et al. 2023) did not state a definition, and the third trial (Fares et al. 2014) reported a composite of PPCs: individual incidences of pneumonia, pleural effusions and ARDS (acute respiratory distress syndrome). These were all included in the meta-analysis of PPCs. None of these trials specified a time limit for recording these complications. The meta-analysis (Fig. 3) suggests TEA may reduce the risk of a composite of PPCs (*RR* 3.88; 95% *CI* 1.98–7.61), although this is of lower certainty due to some risk of bias and differences in definition of composites of PPCs. The *I*^2^ and *χ*^2^ tests suggest little heterogeneity, although there is some uncertainty of these values as the number of studies and sample sizes is small.

### Postoperative pain scores

Some studies reported only pain scores (Zhu et al. 2020; Wang et al. 2019; Maghsoudloo et al. 2023; Wang et al. 2017; Yokoyama et al. 2005; Liu and Wang 2015) and did not specify rest and dynamic pain scores. In this case, these pain scores were included in the meta-analyses for rest pain scores. Nearly all pain scores were stated as the VAS (visual analogue score), apart from one study which used the box scale and was still included in the pain meta-analyses (Yokoyama et al. 2005). Some dynamic pain scores did not record their dynamic activity or had different activities across trials. All but three trials did not report numerical data for their pain scores and only had graphical data (Li et al. 2019; Wang et al. 2019; Maghsoudloo et al. 2023).

**Fig. 4:**
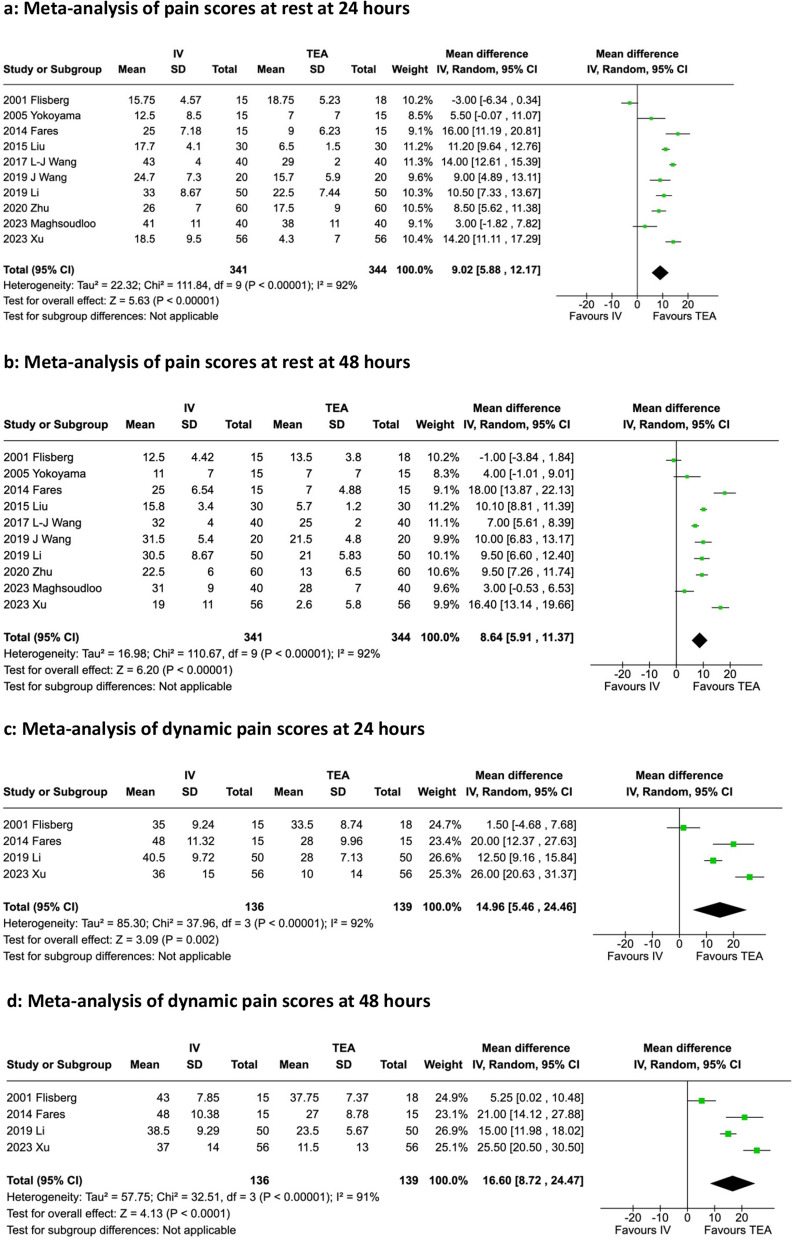
**a** Meta-analysis of pain scores at rest at 24 h. **b** Meta-analysis of pain scores at rest at 48 h. **c** Meta-analysis of dynamic pain scores at 24 h. **d** Meta-analysis of dynamic pain scores at 48 h

Rest pain was meta-analysed at 24 and 48 h (Fig. 4a and b). All trials but one favoured TEA. Both meta-analyses suggest a significant reduction in pain scores in the TEA group regarding rest pain at 24 (*MD* 9.02; 95% *CI* 5.88–12.17) and 48 h (*MD* 8.64; 95% *CI* 5.91–11.37).

Four of 10 trials measured dynamic pain scores at 24 and 48 h; the meta-analyses were displayed in Fig. 4c and d. The summary measures again suggested a significant reduction in pain scores in the TEA group at 24 (*MD* 14.96; 95% *CI* 5.46–24.46) and 48 h (*MD* 16.60; 95% *CI* 8.72–24.47), with a larger effect but wider confidence interval in comparison to the rest pain meta-analyses. The mean difference (MD) is measured in millimetre of the 0–100-mm visual analogue score (VAS). All pain score meta-analyses suggest considerable heterogeneity when assessing their *I*^2^ and *χ*^2^ tests.

### Postoperative nausea and vomiting

**Fig. 5:**
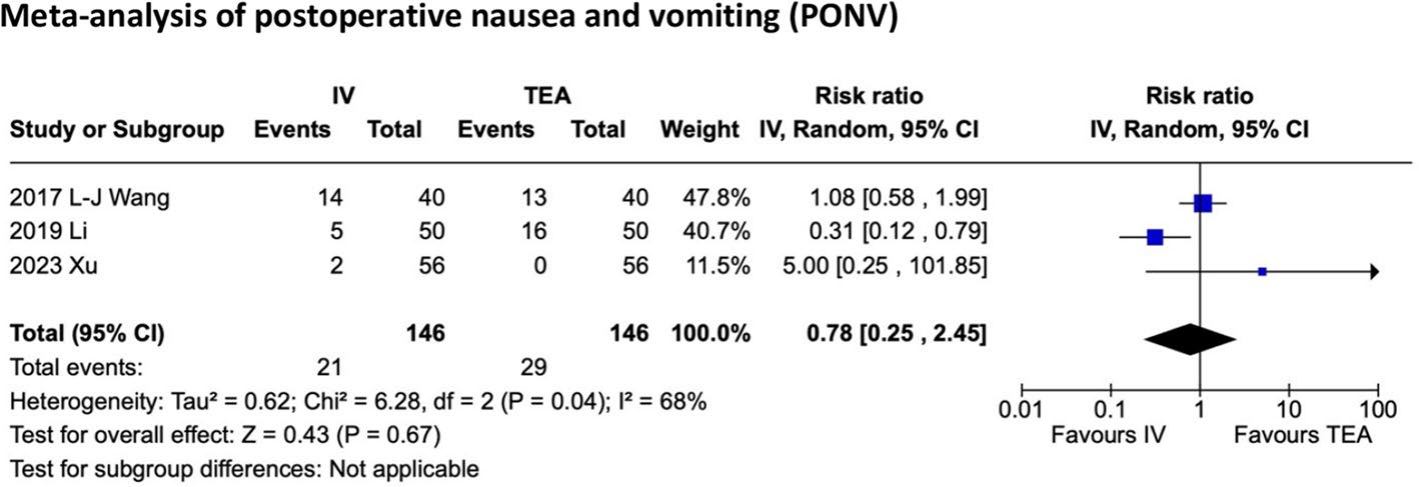
Meta-analysis of postoperative nausea and vomiting (PONV)

Regarding PONV, only three trials reported data, no definitions or time limits were given by any trial and two trials recorded nausea and vomiting as a single event (Xu et al. 2023; Li et al. 2019). One trial recorded them as two separate events (Wang et al. 2017), which were combined into a single event by addition, in order to include it in the meta-analysis (Fig. 5). This meta-analysis did not suggest any difference in reduction of PONV rates by either method of analgesia and should be considered of low certainty.

### Other outcomes

There was not enough data to allow meta-analysis of length of hospital stay. Only two trials recorded total hospital stay, both without standard deviations and measuring time to the nearest day (Xu et al. 2023; Flisberg et al. 2001). Other trials’ measurements varied including critical care unit stay, postoperative care unit stay and pre- and postoperative hospital stay with different units of measuring time.

Only one trial recorded technical failure of epidurals (Flisberg et al. 2001). This trial recorded 4 failures of epidural analgesia in 18 participants (22%), and the definition of failure and causes was not recorded. Assuming the definition of failure was similar to other trials, this would be in keeping with failure rates reported in the current literature (27–32%) (Hermanides et al. 2012).

## Discussion

This meta-analysis provides evidence for a significant reduction in a composite of PPCs in patients receiving TEA compared with IV opioids. However, overall certainty of evidence is low. Two of the three trials assessing PPCs did not adhere to a standardised definition, and one trial measured composites of PPCs such as pneumonia, atelectasis or pleural effusion. A limitation of composite measurements is the lack of clarity as to which component is different. The solution to this is to use core outcome sets (such as those from the StEP — COMPAC group) with standardised endpoints, creating more comparable data for future meta-analyses (Myles et al. 2016; Boney et al. 2022). Also, this result was on the basis of a small number of small trials, with one trial being judged as a high risk of bias.

Regarding non-epidural regional techniques, we found only one trial directly comparing these to thoracic epidural analgesia. Xu et al. (2023) had 3 groups with 56 patients in each group and showed a similar 5 and 7 PPCs in its TEA group versus its PVB/TAP group, contrasting to the larger 17 PPCs in its IV group (Xu et al. 2023). Therefore, this combined single-shot paravertebral and transversus abdominus plane block technique (PVB/TAP) shows promise for the future but would benefit from a larger body of evidence. A disadvantage is that this technique would require expertise and time for two separate procedures; an advantage would be no requirement for running a postoperative neuraxial local anaesthetic infusion with its associated risks.

Regarding the secondary outcome of pain scores, this review shows a significant reduction in pain scores for patients receiving TEA compared to intravenous analgesia. This significance is displayed at 24 and 48 h postoperatively, at rest and during dynamic movement. This is in contrast to the two previous systematic reviews in 2017 and 2018 which did not show a significant difference in pain scores, probably due to a paucity of data at that time (Visser et al. 2017; Hughes et al. 2018). But this is in agreement with a 2024 network meta-analysis of 14 trials, which also suggests a statistically significant reduction in pain scores with epidural versus systemic opioids (Ramjit et al. 2024). However, our results may only be clinically significant for the dynamic pain scores, which have a MD above that of the 10-mm minimum clinically important difference suggested in the literature (Myles et al. 2017). The single non-epidural regional technique in our review (PVB/TAP group) showed higher pain scores in its trial than the TEA group but lower pain scores than the IV group, with varying levels of significance, and poorer pain control as time progressed (Xu et al. 2023).

We were unable to compare patient-controlled epidural analgesia (PCEA) versus continuous epidural analgesia in this review, as only two trials ran continuous epidural regimens (Maghsoudloo et al. 2023), (Fares et al. 2014). There was also a large amount of methodological diversity within trials that allowed epidural boluses, some being clinician bolus only (not PCEA) (Flisberg et al. 2001; Yokoyama et al. 2005). Bolus volumes varied, there were differences in concentration and type of local anaesthetic and some trials had unspecified lockout times (Flisberg et al. 2001; Wang et al. 2017). Epidurals were sited at different thoracic vertebral levels with one trial siting two thoracic epidurals (Yokoyama et al. 2005).

Other limitations include the small numbers of participants in some trials with no large clinical effectiveness trials. Small RCTs can overestimate treatment effects in the real world (Dechartres et al. 2024). There was methodological diversity in the inclusion and exclusion criteria, populations were from different countries and there were differences in surgical approaches. Nearly all included studies used an open approach (large abdominal incision), with one study using a laparoscopic approach. Although there is a lack of evidence regarding analgesic strategies in laparoscopic surgery, omitting this study does not significantly change the results of the meta-analyses. These differences may have contributed to the high statistical heterogeneity in the pain meta-analyses.

Trials had little data on morbidity which was also poorly defined, and many did not assess pain after 48 h. However, within the 48-h postsurgical timeframe, pain scores were well reported, with nearly all trials using a visual analogue scale (VAS) at standardised time intervals. Pain measurement with the VAS is a validated, subjective measure in acute pain which is well understood by patients (Delgado et al. 2024; Haefeli and Elfering 2006). Its measurement at rest and movement at 24 h is also considered a key patient-reported outcome measure (Myles et al. 2018).

Regarding the limitations of this systematic review process, we did not search for trials with non-epidural regional techniques, unless they included epidural analgesia as a comparator group. This was to avoid the bias associated with indirect comparisons within a network meta-analysis (Feenstra et al. 2023). We searched three large databases, but others were omitted. Regarding the data extraction process, the conversion of graphical data to numerical data using online software was required, which does not have perfect accuracy. One trial did not report its standard deviation for pain scores, and this was imputed in order for inclusion in the meta-analyses (Fares et al. 2014). Lastly, a lack of data for less invasive surgical techniques and non-epidural regional techniques is a limitation of this review. However, these surgical techniques are not yet developed at many centres, are not available for all levels of disease progression and have not yet shown clear short- and long-term benefits (Jebril et al. 2024).

This review has the benefit of including recent evidence and being restricted to oesophagectomy patients only, who have very specific analgesic requirements, compared to older systematic reviews which included data from non-randomised trials (Visser et al. 2017) and data from (non-oesophagectomy) gastric surgery patients (Hughes et al. 2018). It is the first systematic review to interrogate respiratory outcomes, albeit a composite, and it is the first to show evidence to support a reduction in a composite of PPCs with TEA versus intravenous analgesia. It also supports the recent network meta-analysis by Ramjit et al., by showing a reduction in pain scores with TEA versus intravenous analgesia (Ramjit et al. 2024). Contextualising this, our systematic review can support clinicians in utilising thoracic epidural analgesia for reducing pain and PPCs. It can aid discussions in preoperative counselling, shared decision-making and during perioperative risk stratification and planning of postoperative care.

## Conclusions

This meta-analysis provides evidence that TEA should currently remain the gold standard analgesic technique for reducing pain after elective oesophagectomy. It is also the first review to provide evidence that TEA reduces a composite of PPCs following oesophagectomy surgery, although this conclusion is of low certainty. Future trials are needed to compare TEA administration techniques, including PCEA. Non-epidural regional analgesic techniques should also be considered for future research. Trials must include more recent laparoscopic and minimally invasive surgical approaches, since the benefit and risk profile of TEA may not be generalised to these patient groups. Appropriate powering to detect clinical effectiveness is required, as is the use of core outcome sets with standardised endpoints (Myles et al. 2016).

## Data Availability

No datasets were generated or analysed during the current study.

## Appendix

### Search Criteria

1# (o)esophagectom* OR (o)esophagogastric resection OR (o)esophageal surger* OR Ivor Lewis.

AND

2# epidural*

AND

3# Cochrane Highly Sensitive Search Strategy for RCTs (Lefebvre et al. 2023)

Cochrane Highly Sensitive Search Strategy for identifying randomized trials in MEDLINE: sensitivity-maximizing version (2008 revision); **PubMed format**

#1 randomized controlled trial [pt]

#2 controlled clinical trial [pt]

#3 randomized [tiab]

#4 placebo [tiab]

#5 drug therapy [sh]

#6 randomly [tiab]

#7 trial [tiab]

#8 groups [tiab]

#9 #1 OR #2 OR #3 OR #4 OR #5 OR #6 OR #7 OR #8

#10 animals [mh] NOT humans [mh]

#11 #9 NOT #10

Cochrane Highly Sensitive Search Strategy for identifying randomized controlled trials in Embase: (2020 revision); **Embase.com format**

#1 ‘randomized controlled trial’/de

#2 ‘controlled clinical trial’/de

#3 random*:ti,ab,tt

#4 ‘randomization’/de

#5 ‘intermethod comparison’/de

#6 placebo:ti,ab,tt

#7 (compare:ti,tt OR compared:ti,tt OR comparison:ti,tt)

#8 ((evaluated:ab OR evaluate:ab OR evaluating:ab OR assessed:ab OR assess:ab) AND (compare:ab OR compared:ab OR comparing:ab OR comparison:ab))

#9 (open NEXT/1 label):ti,ab,tt

#10 ((double OR single OR doubly OR singly) NEXT/1 (blind OR blinded OR blindly)):ti,ab,tt

#11 ‘double blind procedure’/de

#12 (parallel NEXT/1 group*):ti,ab,tt

#13 (crossover:ti,ab,tt OR ‘cross over’:ti,ab,tt).

#14 ((assign* OR match OR matched OR allocation) NEAR/6 (alternate OR group OR groups OR intervention OR interventions OR patient OR patients OR subject OR subjects OR participant OR participants)):ti,ab,tt

#15 (assigned:ti,ab,tt OR allocated:ti,ab,tt)

#16 (controlled NEAR/8 (study OR design OR trial)):ti,ab,tt

#17 (volunteer:ti,ab,tt OR volunteers:ti,ab,tt)

#18 ‘human experiment’/de

#19 trial:ti,tt

#20 #1OR#2OR#3OR#4OR#5OR#6OR#7OR#8OR#9OR#10OR#11OR#12OR#13 OR #14 OR #15 OR #16 OR #17 OR #18 OR #19

#21 (((random* NEXT/1 sampl* NEAR/8 (‘cross section*’ OR questionnaire* OR survey OR surveys OR database or databases)):ti,ab,tt) NOT (‘comparative study’/de OR ‘controlled study’/de OR ‘randomised controlled’:ti,ab,tt OR ‘randomized controlled’:ti,ab,tt OR ‘randomly assigned’:ti,ab,tt))

#22 (‘cross‐sectional study’/de NOT (‘randomized controlled trial’/de OR ‘controlled clinical study’/de OR ‘controlled study’/de OR ‘randomised controlled’:ti,ab,tt OR ‘randomized controlled’:ti,ab,tt OR ‘control group’:ti,ab,tt OR ‘control groups’:ti,ab,tt))

#23 (‘case control*’:ti,ab,tt AND random*:ti,ab,tt NOT (‘randomised controlled’:ti,ab,tt OR ‘randomized controlled’:ti,ab,tt))

#24 (‘systematic review’:ti,tt NOT (trial:ti,tt OR study:ti,tt))

#25 (nonrandom*:ti,ab,tt NOT random*:ti,ab,tt)

#26 ‘random field*’:ti,ab,tt

#27 (‘random cluster’ NEAR/4 sampl*):ti,ab,tt

#28 (review:ab AND review:it) NOT trial:ti,tt

#29 (‘we searched’:ab AND (review:ti,tt OR review:it))

#30 ‘update review’:ab

#31 (databases NEAR/5 searched):ab

#32 ((rat:ti,tt OR rats:ti,tt OR mouse:ti,tt OR mice:ti,tt OR swine:ti,tt OR porcine:ti,tt OR murine:ti,tt OR sheep:ti,tt OR lambs:ti,tt OR pigs:ti,tt OR piglets:ti,tt OR rabbit:ti,tt OR rabbits:ti,tt OR cat:ti,tt OR cats:ti,tt OR dog:ti,tt OR dogs:ti,tt OR cattle:ti,tt OR bovine:ti,tt OR monkey:ti,tt OR monkeys:ti,tt OR trout:ti,tt OR marmoset*:ti,tt) AND ‘animal experiment’/de)

#33 (‘animal experiment’/de NOT (‘human experiment’/de OR ‘human’/de))

#34 #21OR#22OR#23OR#24OR#25OR#26OR#27OR#28OR#29OR#30OR#31OR #32 OR #33

#35 #20 NOT #34

## Abbreviations

PROSPERO: International Prospective Register of Systematic Reviews
PRISMA: Preferred Reporting Items for Systematic reviews and Meta-Analyses
PICOS: Population, Intervention, Comparison, Outcomes and Study design framework for research projects
PPC: Postoperative pulmonary complications
EPCO: European Perioperative Clinical Outcomes
VAS: Visual analogue scale
PONV: Postoperative nausea and vomiting
MEDLINE: Medical Literature Analysis and Retrieval System Online
EMBASE: Excerpta Medica Database
CENTRAL: Cochrane Central Register of Controlled Trials
HSSS: Cochrane Highly Sensitive Search Strategy filter
RevMan: Review Manager
CEBM: Centre for Evidence-Based Medicine
TEA: Thoracic epidural analgesia
IV: Intravenous
PCA: Patient-controlled analgesia
PCEA: Patient-controlled epidural analgesia
SC: Subcutaneous
TAP: Transversus abdominus plane
PVB: Paravertebral block
ASA: American Society of Anesthesiologists
COPD: Chronic obstructive pulmonary disease
BMI: Body mass index
NSAID: Nonsteroidal anti-inflammatory drug
NYHA: New York Heart Association heart failure grade
CKD: Chronic kidney disease
LOS: Length of stay in hospital
RCT: Randomised controlled trial
NR: Not reported
n/a: Not applicable
CONSORT: Consolidated Standards of Reporting Trials
MD: Mean differences
SD: Standard deviation
RR: Risk ratio
CI: Confidence interval
ARDS: Acute respiratory distress syndrome
StEP: Standardised Endpoints in Perioperative Medicine
COMPAC: Core Outcome Measures for Perioperative and Anaesthetic Care
CINAHL: Cumulative Index to Nursing and Allied Health Literature database
LILACs: Latin American and Caribbean Health Sciences Literature database
